# Rice Cultivation Duration Drives Soil Organic Carbon Stabilization in Saline–Alkaline Paddy Soils via Mineral-Associated Organic Carbon Accumulation and Biological Pathways

**DOI:** 10.3390/biology15171452

**Published:** 2026-08-25

**Authors:** Fanbing Xu, Minghui Wang, Ziyue Lu, Yuanbo Xie, Liming Tian, Caixia Lv, Xiwen Zhang, Yuhang Song, Xiao Yao, Hongjian Zhang, Dan Zhang

**Affiliations:** College of Landscape Architecture, Changchun University, Changchun 130022, China; 241201420@mails.ccu.edu.cn (F.X.); 241203452@mails.ccu.edu.cn (M.W.); 221201410@mails.ccu.edu.cn (Z.L.); 241202441@mails.ccu.edu.cn (Y.X.); 251203475@mails.ccu.edu.cn (L.T.); 251203461@mails.ccu.edu.cn (C.L.); 251203479@mails.ccu.edu.cn (X.Z.); 231201437@mails.ccu.edu.cn (Y.S.); 231203459@mails.ccu.edu.cn (X.Y.); 231201432@mails.ccu.edu.cn (H.Z.)

**Keywords:** saline–alkaline paddy soil, chronosequence, soil organic carbon fraction, mineral-associated organic carbon, Fourier transform infrared spectroscopy, carbon sequestration

## Abstract

Continuous paddy reclamation is a promising strategy for restoring sodic lands, yet the exact mechanisms driving soil organic carbon (SOC) stabilization remain insufficiently quantified. Here, we investigated SOC dynamics across a 12-year rice cultivation chronosequence (0, 2, 5, 10, and 12 years) at 0–20 cm and 20–40 cm depths in western Jilin Province, China. Successive rice cultivation progressively alleviated saline–alkaline stress, with electrical conductivity (EC) decreasing by 70.11% in topsoil after 12 years, establishing a stabilized soil environment by years 10–12. Concurrently, topsoil SOC and total nitrogen (TN) increased by 63.09% and 26.02%, respectively. This physicochemical amelioration triggered a directional carbon transformation: while particulate organic carbon (POC) accumulated in early stages, mineral-associated organic carbon (MAOC) dominated medium-term storage, expanding by 147.12% in topsoil and elevating its share of SOC. Fourier transform infrared (FTIR) spectroscopy confirmed a shift toward molecular structural persistence, marked by increased aromaticity and hydrophobicity. Partial least squares path modeling (PLS-PM) demonstrated that cultivation duration directly drove soil stability (*β* = 0.94, *p* < 0.001), operating through a hierarchical cascade where management-induced stress reduction enhanced soil enzyme activity and microbial processing, thereby accelerating the conversion of labile plant inputs into mineral-protected MAOC. Overall, this study quantifies the pivotal role of paddy management in driving organo-mineral protection and chemical persistence, providing a mechanistic framework for carbon sequestration in degraded agroecosystems.

## Simple Summary

Soil salinization severely reduces agricultural productivity and soil carbon sequestration potential. Based on a 12-year rice cultivation chronosequence in saline–alkaline paddy soils of western Jilin, China, we examined soil organic carbon stabilization dynamics and underlying mechanisms. Continuous rice cropping markedly alleviated saline–alkali stress, decreasing topsoil EC by 70.11% and increasing soil organic carbon (SOC) by 63.09%. SOC was progressively converted to stable pools, with topsoil mineral-associated organic carbon (MAOC) increasing by 147.12%. This shift was driven by improved soil physicochemical conditions and enzyme-mediated microbial processes, which facilitated the formation of mineral-protected, aromatic-stabilized organic carbon. Our results confirm that rice cultivation serves as an effective practice to restore degraded saline–alkaline soils and enhance stable soil carbon sinks.

## 1. Introduction

With the continuous increase in greenhouse gases, global warming has become a global consensus. As the largest carbon pool in terrestrial ecosystems, soil exerts a pivotal regulatory role in the global carbon balance. Studies have shown that the global soil organic carbon (SOC) stock is 1300–2000 Pg, markedly exceeding the total carbon storage of the vegetation and the atmospheric carbon pool [1]. Farmland ecosystems exhibit highly dynamic component of the terrestrial carbon cycle, with their carbon stock accounting for 8–10% of the global soil carbon stock [2]. The dynamics of farmland SOC not only directly affect soil fertility and crop yield [3] but also feedback to climate change by altering atmospheric carbon dioxide (CO_2_) concentration [4]. Thus, exploring the mechanism underlying SOC sequestration in farmland soils is of great significance for enhancing soil fertility and maintaining the sustainable development of agriculture.

Against this background, soil salinization–alkalization, driven by anthropogenic and natural factors, has become an urgent global environmental issue. Persistent accumulation of water-soluble salts induces excessive soil salt enrichment and degradation [5,6], directly causing drastic land productivity decline characterized by low crop emergence, stunted growth and sharp yield reduction, thus threatening agricultural production, ecological security and socio-economic development [7]. The Western region of Jilin Province is a typical sodic saline–alkaline area in Northeast China, with salinized–alkalized soils covering approximately 1.67 × 10^6^ hm^2^ (94% moderate–severe), severely restricting sustainable agricultural development [8]. Though salt-sensitive, rice has a certain alkali tolerance, and its root exudates facilitate soil acidification to alleviate saline–alkaline constraints [9]. A rice cultivation-based saline–alkaline soil improvement model has been widely promoted in the western region of Jilin province, with saline–alkaline paddy fields reaching 8 × 10^5^ hm^2^ by the early 21st century (3 × 10^5^ hm^2^ converted from moderate–severe saline–alkaline soils) [10]. Additionally, biochar combined with rice cultivation enhances saline–alkaline soils’ carbon sink capacity by over 50%, meanwhile improving soil structure and reducing salinity [11]. Therefore, studying SOC variation patterns in saline–alkaline paddy soil under different rice cultivation durations and revealing its stability and sequestration mechanisms is crucial for enhancing saline–alkaline land carbon sink capacity.

Farmland SOC dynamics, jointly regulated by anthropogenic management and natural conditions, directly modulate agricultural productivity and the global carbon cycle. Tillage activities significantly affect SOC accumulation and loss by disturbing soil structure, regulating soil moisture and aeration conditions, and altering the physical protection of soil organic carbon via aggregate stability. Continuous tillage reduces SOC content, while reasonable tillage combined with carbon input management elevates SOC levels within five years [12]. Cultivation duration exerts divergent effects on SOC across rice-growing regions [13]. For example, subtropical rice areas showed rapid and sustainable SOC accumulation from never being cultivated to 55 years of cultivation [14]. Jilin’s saline–alkaline paddy fields showed increasing SOC with extended cultivation, which presented and stabilized after 5–7 years cultivation; Northeast China’s dark brown soil paddies witnessed significant SOC rise in 5–20 years of reclamation followed by a slowdown [15], while black soil paddies exhibited a “decrease-then-increase” SOC trend with a sharp drop in the first 3 years of dryland-to-paddy conversion and gradual recovery over 3–25 years [16,17]. Tropical alkaline soils has lower MAOM carbon storage than acidic soils, as Ca^2+^ and Mg^2+^ weaken organic carbon–mineral interactions via cation bridges [18]. These variations highlight that extending rice cultivation duration directly drives the chronological evolution of soil physicochemical properties—such as the mitigation of sodium ion stress, optimization of soil structure, and stimulation of root–microbial interactions—which sequentially dictate the physical protection via aggregate formation and chemical stabilization through mineral–organic associations.

SOC is a heterogeneous mixture of labile, unstable and easily oxidizable fractions whose composition and distribution are highly sensitive to environmental changes and agricultural management. Ref. [19], via Fourier transform infrared spectroscopy (FTIR) spectroscopy analysis, found that extended reclamation duration increased aromatic-C (1630 cm^−1^) but decreased aliphatic-C (2920 cm^−1^) in arid valley soils, indicating enhanced SOC stability. Physical fractionation is widely used to explore SOC dynamic mechanisms, and this method has advanced from Six et al.’s early aggregate density combined fractionation (focusing on aggregate protection) to the current simplified dichotomy of particulate organic carbon (POC) and mineral-associated organic carbon (MAOC) based on turnover and stability [20]. SOC fractions are more sensitive to cultivation duration and management than SOC and can reflect soil quality [21]. POC, a labile fraction of semi-decomposed plant residues, is highly disturbance-sensitive and serves as an early indicator of SOC changes [22]. MAOC, a stable pool bound to fine particles from organic matter decomposition, has strong chemical inertness [23,24]. POC usually increases in early cultivation (0–10 years) then decreases, while MAOC increases with extended cultivation [25]. Previous study has reported that crop planting increased the MAOC/POC ratio and SOC stability in coastal saline–alkaline soils [26]. However, current studies on saline–alkaline paddy SOC mostly focus on fertilization and agronomic practices, with limited research on how rice cultivation duration affects SOC fractions and their stability in western Jilin’s saline–alkaline paddy soils.

Therefore, saline–alkaline paddy soils in western Jilin Province were selected as the study system. Uncultivated saline–alkaline soil served as the control (CK), and soils with rice cultivation histories of 2, 5, 10, and 12 years were used to establish a cultivation chronosequence. Based on the historical management of these soils, we formulated three interconnected hypotheses: (1) prolonged rice cultivation progressively alleviates saline–alkaline stress and improves soil fertility; (2) increasing cultivation duration enhances overall soil organic carbon (SOC) accumulation; (3) the long-term stabilization of SOC is primarily driven by the promotion of mineral-associated organic carbon (MAOC) formation and distinct alterations in the functional-group composition of SOC. Accordingly, this study aimed to (1) characterize changes in soil salinity and fertility across the rice cultivation chronosequence; (2) quantify the effects of cultivation duration on SOC fractions, including POC and MAOC, and their chemical characteristics; and (3) identify the potential mechanisms underlying SOC stabilization in saline–alkaline paddy soils. The results of this study not only can provide direct scientific basis for the precision improvement of saline–alkaline soils, sustainable rice cultivation, and nutrient management in western Jilin Province, but can also offer important theoretical support and reference for the enhancement of carbon sink capacity and the assessment of agricultural carbon sequestration potential in the context of addressing climate change.

## 2. Materials and Methods

### 2.1. Study Area

This study area is located at Kenong Guye Base in Dongfanjia Village, Yongping Township, Fuyu City, Jilin Province (44°50′–45°30′ N, 124°40′–126°12′ E). Situated in the hinterland of the Songnen Plain, the area has an average altitude of 130–140 m and is characterized by a temperate continental monsoon climate. The annual average temperature ranges from 4 to 5 °C, with an annual precipitation of 350–500 mm (mainly concentrated in summer). The frost-free period lasts approximately 140–150 days, and the area enjoys sufficient sunlight [27]. Owing to combined effects of the regional geological setting, climatic conditions, and biogeochemical cycling, calcic soils typical of the arid steppe zone are widely distributed in western Jilin Province. According to regional soil classification records, the dominant soil groups in Fuyu City are Phaeozems and Chernozems [28]. However, the low-lying topography, poor drainage, intense evaporation, and agricultural activities have resulted in varying degrees of soil salinization and sodification, with severe saline–sodic degradation occurring in some areas [29].

### 2.2. Soil Sampling

In this study, saline–alkaline paddy soils with rice cultivation durations of 2, 5, 10, and 12 years were selected as treatment groups, and uncultivated saline–alkaline soil was used as the control (CK). After rice harvest, one representative field was sampled for each treatment. Five sampling points were arranged along an S-shaped transect in each field, and soils were collected from the 0–20 cm and 20–40 cm soil layers. For each depth, the five subsamples were thoroughly mixed to form one composite sample. After removing impurities such as plant roots and stones, the collected soil samples were divided into two parts. One part was air-dried, ground, and sieved for the determination of soil physicochemical properties, soil ion content, and soil organic carbon fractions. The other part was stored in a refrigerator at 4 °C for the analysis of soil enzyme activity; a small subsample of this portion was preserved in an ultra-low-temperature refrigerator at −80 °C for the determination of microbial community composition.

### 2.3. Determination of Soil Properties, Soil Ion Content, Soil Organic Carbon Fractions, and Soil Enzymes

Soil physicochemical properties were determined according to the methods described in *Soil Agrochemical Analysis* edited by Bao Shidan [30]. Soil pH and electrical conductivity (EC) were measured in soil–water suspensions at ratios of 1:2.5 and 1:5 (*w*/*v*), respectively. Soil bulk density (SBD) and soil water content (SWC) were determined using the core and oven-drying methods, respectively. For the determination of total nitrogen (TN), soil samples were digested with concentrated H_2_SO_4_ and a mixed catalyst following the standard Kjeldahl method. For total phosphorus (TP), the samples were digested with a mixture of concentrated H_2_SO_4_ and H_2_O_2_. Following their respective digestion procedures, the nitrogen and phosphorus concentrations in the digests were both quantified colorimetrically using a continuous flow analyzer (SEAL AA3, SEAL Analytical, Norderstedt, Germany). Nitrate nitrogen (NO_3_^−^-N) and ammonium nitrogen (NH_4_^+^-N) were extracted with 2 mol L^−1^ KCl (soil–solution = 1:5, *w*/*v*), followed by determination using ultraviolet spectrophotometry and the indophenol blue colorimetric method, respectively, with a UV-Vis spectrophotometer (UVmini-1280, Shimadzu Corporation, Kyoto, Japan). Water-soluble K^+^, Na^+^, Ca^2+^, Mg^2+^, Cl^−^, CO_3_^2−^, HCO_3_^−^, and SO_4_^2−^ were determined in a 1:5 soil–water extract using standard flame photometry, titration, and complexometric methods. Water-soluble ions (K^+^, Na^+^, Ca^2+^, Mg^2+^, Cl^−^, CO_3_^2−^, HCO_3_^−^, and SO_4_^2−^) were extracted using a 1:5 (*w*/*v*) soil–water ratio. K^+^ and Na^+^ were determined using a flame photometer (FP6450, INESA Analytical Instrument Co., Ltd., Shanghai, China). Ca^2+^ and Mg^2+^ were measured by EDTA complexometric titration. Cl^−^ was determined by silver nitrate titration (Mohr’s method). CO_3_^2−^ and HCO_3_^−^ were quantified by standard acid–base titration. SO_4_^2−^ was determined by EDTA indirect titration.

POC and MAOC were separated using the sodium hexametaphosphate dispersion method. Specifically, soil samples were dispersed with 5 g L^−1^ sodium hexametaphosphate and shaken, followed by wet-sieving to isolate POC (>53 μm) and MAOC (<53 μm). The organic carbon content in each fraction, as well as SOC, was determined using the potassium dichromate external heating–ferrous titration method.

Soil cellulase (CEL) and invertase (INV) activities were determined using the 3,5-dinitrosalicylic acid colorimetric method [31]. Catalase (CAT) activity was determined using the potassium permanganate volumetric method [32]. Polyphenol oxidase (PPO) activity was determined using the catechol iodine titration method [33]. *β*-glucosidase (GLU) activity was determined using the nitrophenol colorimetric method [34]. All absorbance measurements for enzyme activities were performed using a FLUOstar Omega microplate reader (BMG LABTECH GmbH, Ortenberg, Germany).

All analytical-grade chemical reagents used in the above tests were purchased from Sinopharm Chemical Reagent Co., Ltd. (Shanghai, China).

### 2.4. Determination of Infrared Spectral Characteristics

Air-dried samples of the separated soil organic carbon fractions were analyzed by Fourier transform infrared spectroscopy. Each sample (0.005 g) was mixed with spectroscopic-grade KBr at a mass ratio of 1:100, thoroughly ground in an agate mortar under an infrared lamp, and pressed into a transparent pellet at 15 MPa for 1 min. FTIR spectra were acquired using a Nicolet iS5 spectrometer (Thermo Fisher Scientific, Waltham, MA, USA) over the range of 4000–400 cm^−1^ at a resolution of 4 cm^−1^, with 32 scans averaged per spectrum. A pure KBr pellet was used for background collection. Spectra were processed using OMNIC 9.0 software (Thermo Fisher Scientific, Waltham, MA, USA), including atmospheric background subtraction, conversion from transmittance to absorbance, baseline correction, and vector normalization. Based on spectral absorption peak characteristics, the spectra were divided into 6 functional group regions, including alcoholic and phenolic hydroxyl groups (O-H, 3680~3200 cm^−1^), aliphatic hydrocarbons (C-H, 3000~2800 cm^−1^ and 1470~1385 cm^−1^), aromatic alkenes (C=C, 1845~1490 cm^−1^), aromatic hydrocarbons (C-H, 930~840 cm^−1^), and polysaccharide carboxyl groups (C-O, 1385~930 cm^−1^). Subsequently, specific absorption peak ratios were calculated to determine functional groups proportions [35].

### 2.5. Soils Calculation of Soil Stability Index

SOC stability indices are key properties for characterizing the SOC stability, with higher values representing greater SOC stability. The three indexes are prioritized as AI, A/O-A, and HI. The AI reflects the relative content of aromatic components to aliphatic components in SOC [36,37].

The A/O-A characterizes the relative enrichment of alkyl carbon (the core of aliphatic groups) versus polysaccharide carbon, and the HI indicates the hydrophobicity intensity of SOC [38,39].

(1) $$\mathrm{Aromaticity}\;\mathrm{Index}\;(\mathrm{AI})=\frac{[\mathrm{Aromatic}\;\mathrm{alkenes}\;C=C+\mathrm{Aromatic}\;\mathrm{hydrocarbons}\;C\text{-}H]}{[\mathrm{Aliphatic}\;\mathrm{hydrocarbons}\;C\text{-}H]}$$

(2) $$\mathrm{Alkyl}\;\mathrm{carbon}/\mathrm{polysaccharide}\;\mathrm{carbon}\;\mathrm{Index}\;(A/O\text{-}A)=\frac{\left[\mathrm{Aliphatic}\;\mathrm{hydrocarbons}\;C\text{-}H\right]}{\left[\mathrm{Polysaccharide}\;\mathrm{carboxyl}\;\mathrm{groups}\;C\text{-}O\right]}$$

(3) $$\mathrm{Hydrophobicity}\;\mathrm{Index}\;(\mathrm{HI})=\frac{[\mathrm{Aromatic}\;\mathrm{alkenes}\;C=C+\mathrm{Aromatic}\;\mathrm{hydrocarbons}\;C\text{-}H+\mathrm{Aliphatic}\;\mathrm{hydrocarbons}\;C\text{-}H]}{\left[\mathrm{Alcoholic}\;\mathrm{and}\;\mathrm{phenolic}\;\mathrm{hydroxyl}\;\mathrm{groups}\;O\text{-}H+\mathrm{Polysaccharide}\;\mathrm{carboxyl}\;\mathrm{groups}\;C\text{-}O\right]}$$

### 2.6. Data Analysis

Initial data preprocessing was performed using Microsoft Excel 2021. Prior to statistical testing, data normality and homogeneity of variance were verified using the Shapiro–Wilk test and Levene’s test, respectively. Prior to statistical testing, data normality and homogeneity of variance were verified using the Shapiro–Wilk test and Levene’s test, respectively. One-way analysis of variance (ANOVA) was conducted separately for each soil layer to test differences in soil physicochemical properties and soil ion concentrations among rice cultivation durations. When the overall ANOVA was significant, Duncan’s multiple range test was used for post hoc comparisons. Differences between the 0–20 and 20–40 cm soil layers within each cultivation duration treatment were assessed using paired samples *t*-tests. Two-way analysis of variance (two-way ANOVA) was used to evaluate the main and interactive effects of rice cultivation duration and soil depth on soil organic carbon fractions (POC and MAOC). When the interaction between cultivation duration and soil depth was significant, simple effects analyses were performed to compare rice cultivation durations separately within each soil depth, followed by Fisher’s least significant difference (LSD) test for pairwise comparisons. When the interaction was not significant, the main effects of cultivation duration and soil depth were interpreted independently. Statistical significance was accepted at *p* < 0.05. FTIR spectral data were processed with OMNIC Infrared Spectroscopy Software 9.0 (OMNIC 9.0) to generate spectral graphs, and functional group contents were determined according to the characteristics of infrared absorption peaks. Origin 2024 was used for graph plotting. Prior to correlation analysis, the normality of each variable was assessed using the Shapiro–Wilk test. Because the variables were approximately normally distributed, Pearson’s correlation analysis was performed to examine. Correlations were regarded as statistically significant at *p* < 0.05. In the correlation plots, *, **, and *** indicate *p* < 0.05, *p* < 0.01, and *p* < 0.001, respectively. All statistical analyses were conducted in R version 4.4.3, and the correlation plots were generated using the corrplot package (version 0.95).

### 2.7. Partial Least Squares Path Modeling

PLS-PM was performed using the plspm package (version 0.6.0) in R (version 4.4.3) to evaluate the direct and indirect effects of rice cultivation duration on soil organic carbon stability. Rice cultivation duration was specified as the exogenous variable, whereas soil properties, soil ion content, soil enzyme activity, microbial community, and soil stability were specified as latent variables represented by their corresponding observed variables. The structural model was established according to the hypothesized relationships among rice cultivation duration, soil physicochemical properties, microbial processes, and SOC stability. Model performance was evaluated using the coefficient of determination (R^2^) and the goodness-of-fit (GoF > 0.36) index. Indicator reliability was confirmed with outer model loadings > 0.707, and the significance of path coefficients was assessed via bootstrap resampling with 5000 iterations.

## 3. Results

### 3.1. Effects of Rice Cultivation Duration on Soil Properties of Saline–Alkaline Soils

**Figure 1:**
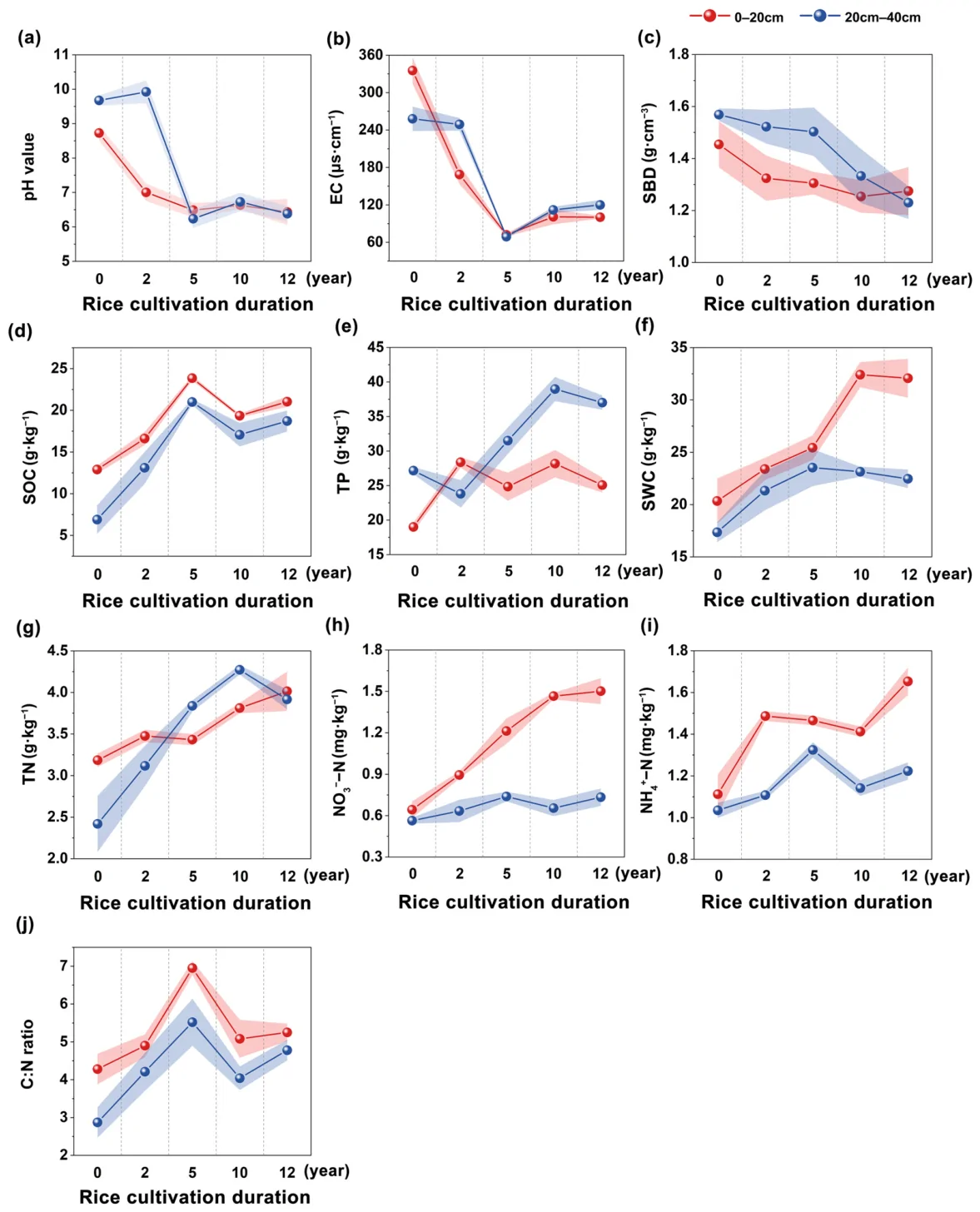
Changes in soil properties of saline–alkaline soils in different soil layers under different rice cultivation durations. (**a**) pH value, (**b**) electrical conductivity, (**c**) soil bulk density, (**d**) soil organic carbon, (**e**) total phosphorus content, (**f**) soil water content, (**g**) total nitrogen content, (**h**) NO_3_^−^−N content, (**i**) NH_4_^+^–N content, (**j**) C:N ratio. Shaded areas represent the standard deviation (SD).

Affected by different rice cultivation durations (2, 5, 10, and 12 years), soil properties (pH value, EC, SBD, SOC, TP, SWC, TN, NO_3_^−^-N, NH_4_^+^-N in different soil layers (0–20 cm, 20–40 cm) underwent significant changes, showing an overall trend of increased nutrient properties and decreased saline–alkaline-related soil properties (Figure 1). Soil pH, EC, and SBD (Figure 1a–c) all showed a decreasing trend with the extension of rice cultivation duration. Among them, EC gradually stabilized after the initial decrease. After 12 years of cultivation, the reduction rates of all properties were significant. Among them, pH decreased by 26.28% and 34.01% in the 0–20 cm and 20–40 cm layers, respectively; SBD decreased by 12.35% and 21.60%, with significant differences among different rice cultivation durations (*p* < 0.05), reflecting progressive salt amelioration and structural loosening induced by paddy management. EC had the most obvious decrease, with reduction rates reaching 70.11% and 53.53%, respectively.

Soil nutrient properties exhibited a consistent stepwise increase followed by stabilization with prolonged cultivation. SOC, TP, and SWC (Figure 1d–f) increased gradually in both soil layers and finally stabilized, indicating cumulative organic matter input and enhanced moisture retention. With SOC and SWC showing significant differences among different rice cultivation durations and soil layers (*p* < 0.05), compared with the control, their contents after 12 years of cultivation increased by 63.09% and 171.55% (SOC), 31.93% and 36.25% (TP), as well as 57.72% and 29.45% (SWC) in the 0–20 cm and 20–40 cm layers, respectively.

As the core indicators of soil nitrogen fertility, TN, NO_3_^−^-N, and NH_4_^+^-N (Figure 1g–i) generally showed increasing trends with rice cultivation duration. After 12 years of cultivation, TN contents increased significantly by 26.02% and 61.94%, while NO_3_^−^-N increased by 133.66% and 30.01%, and NH_4_^+^-N increased by 48.68% and 18.20% in the 0–20 cm and 20–40 cm soil layers, respectively. The soil C:N ratio (Figure 1j) exhibited a unimodal pattern, increasing during the early cultivation stage, reaching a maximum after 5 years, and then declining slightly with prolonged cultivation, although it remained higher than that in the uncultivated soil. This pattern indicates that the relative accumulation rates of SOC and TN differed across the rice cultivation chronosequence.

### 3.2. Effects of Rice Cultivation Duration on Soil Ion Content of Saline–Alkaline Soils

**Figure 2:**
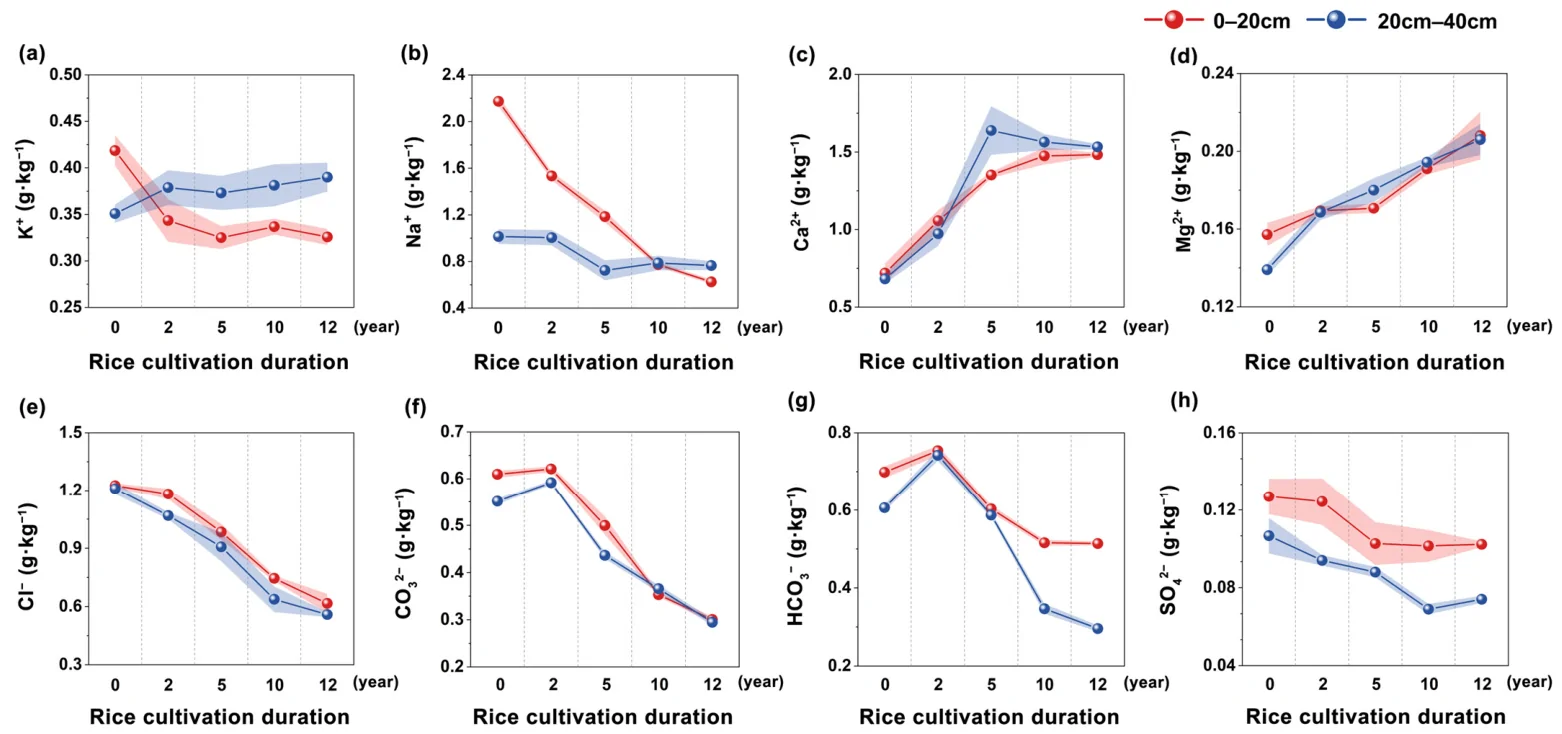
Changes in soil ion content of saline–alkaline paddy soils in different soil layers under different rice cultivation duration. (**a**) K^+^ content, (**b**) Na^+^ content, (**c**) Ca^2+^ content, (**d**) Mg^2+^ content, (**e**) Cl^−^ content, (**f**) CO_3_^2−^ content, (**g**) HCO_3_^−^ content, (**h**) SO_4_^2−^ content. Shaded areas represent the standard deviation (SD).

Soil ion content and dynamics are key properties characterizing soil chemical properties, and the variations of major ions during the saline–alkaline paddy cultivation process were analyzed (Figure 2). Rice cultivation duration significantly altered ion contents, with distinct variation patterns and significant levels across ion species, reflecting active ion leaching and exchange processes driven by paddy management. Among cations, Na^+^ content decreased continuously with extended rice cultivation duration, showing significant differences among different rice cultivation durations and soil layers (*p* < 0.05). After 12 years of rice cultivation, Na^+^ content in the 0–20 cm and 20–40 cm soil layers decreased significantly by 71.24% and 24.54% compared with CK, respectively. In contrast, Ca^2+^ content increased stepwise and stabilized with longer reclamation, with significant differences among durations (*p* < 0.05), rising by 105.95% and 124.78% in the two soil layers after 12 years cultivation relative to CK. Anion contents showed an overall decreasing trend: in the two soil layers, Cl^−^ content decreased by 49.77% and 53.82% after 12 years rice cultivation, with significant differences in the 0–20 cm soil layer (*p* < 0.05). Except for SO_4_^2−^ in the 0–20 cm soil layer, 12-year rice cultivation induced significant differences in Cl^−^, CO_3_^2−^ and HCO_3_^−^ contents compared with CK (*p* < 0.05), pointing to active carbonate neutralization and salt removal processes.

### 3.3. Effects of Rice Cultivation Duration on Soil Organic Carbon Fractions and Functional Group Characteristics of Saline–Alkaline Soils

#### 3.3.1. Effects of Rice Cultivation Duration on the Content of Soil Organic Carbon Fractions in Saline–Alkaline Paddy Soils

**Figure 3:**
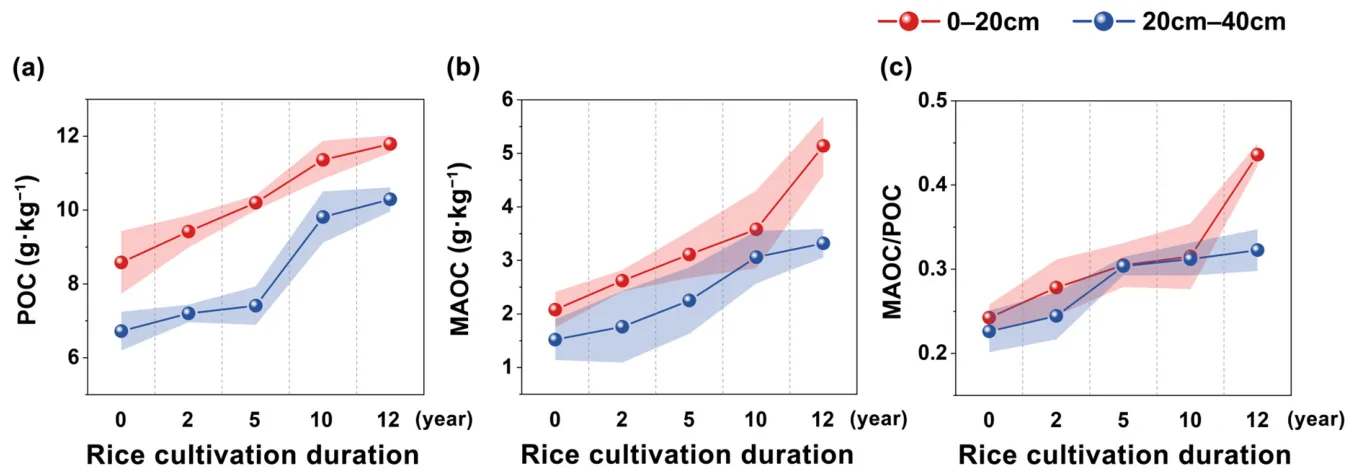
Changes in soil organic carbon fractions. (**a**) POC, (**b**) MAOC, and (**c**) MAOC to POC ratio in saline–alkaline paddy soils in different soil layers under different rice cultivation durations. Shaded areas represent the standard deviation (SD).

Two-way analysis of variance showed that rice cultivation duration and the interactive effect between rice cultivation duration and soil depth had an extremely significant effect on soil POC (*p* < 0.001) (Table 1). As an active intermediate in soil carbon cycling, the content of POC in saline–alkaline paddy soils under different rice cultivation durations showed an increasing trend with the extension of rice planting years, reflecting progressive organic carbon input and accumulation, and have significant differences among different rice cultivation durations (*p* < 0.05) (Figure 3a). After 2, 5, 10, and 12 years of cultivation, the POC content in the 0–20 cm soil layer increased by 9.79%, 18.88%, 32.40%, and 37.41% compared with CK, respectively, while in the 20–40 cm soil layer, it increased by 7.14%, 10.27%, 45.98%, and 53.13%, respectively.

Rice cultivation duration, soil depth, and their interactive effect had an extremely significant effect on soil MAOC (*p* < 0.001) (Figure 3). The MAOC content in both soil layers increased with the extension of rice cultivation duration, pointing to enhanced mineral-associated organic matter stabilization and chemical protection. After 2, 5, 10, and 12 years of cultivation, the MAOC content in the 0–20 cm soil layer increased by 25.96%, 49.52%, 72.12%, and 147.12% compared with CK, respectively, while in the 20–40 cm soil layer, it increased by 15.79%, 48.03%, 101.32%, and 118.42%, respectively. Interlayer differences gradually emerged after 5 years of cultivation (*p* < 0.05) (Figure 3b).

With extended rice cultivation duration, the proportion of MAOC in the SOC pool increased continuously, especially significantly in the 0–20 cm soil layer. With the increase in rice cultivation duration, the ratio of MAOC/POC in the 0–20 cm soil layer increased significantly, demonstrating preferential mineral-associated carbon sequestration in surface soils, while the ratio of MAOC/POC in the 20–40 cm soil layer increased slowly.

#### 3.3.2. Effects of Rice Cultivation Duration on FTIR Functional Group Characteristics of Soil Organic Carbon Fractions in Saline–Alkaline Soils

**Figure 4:**
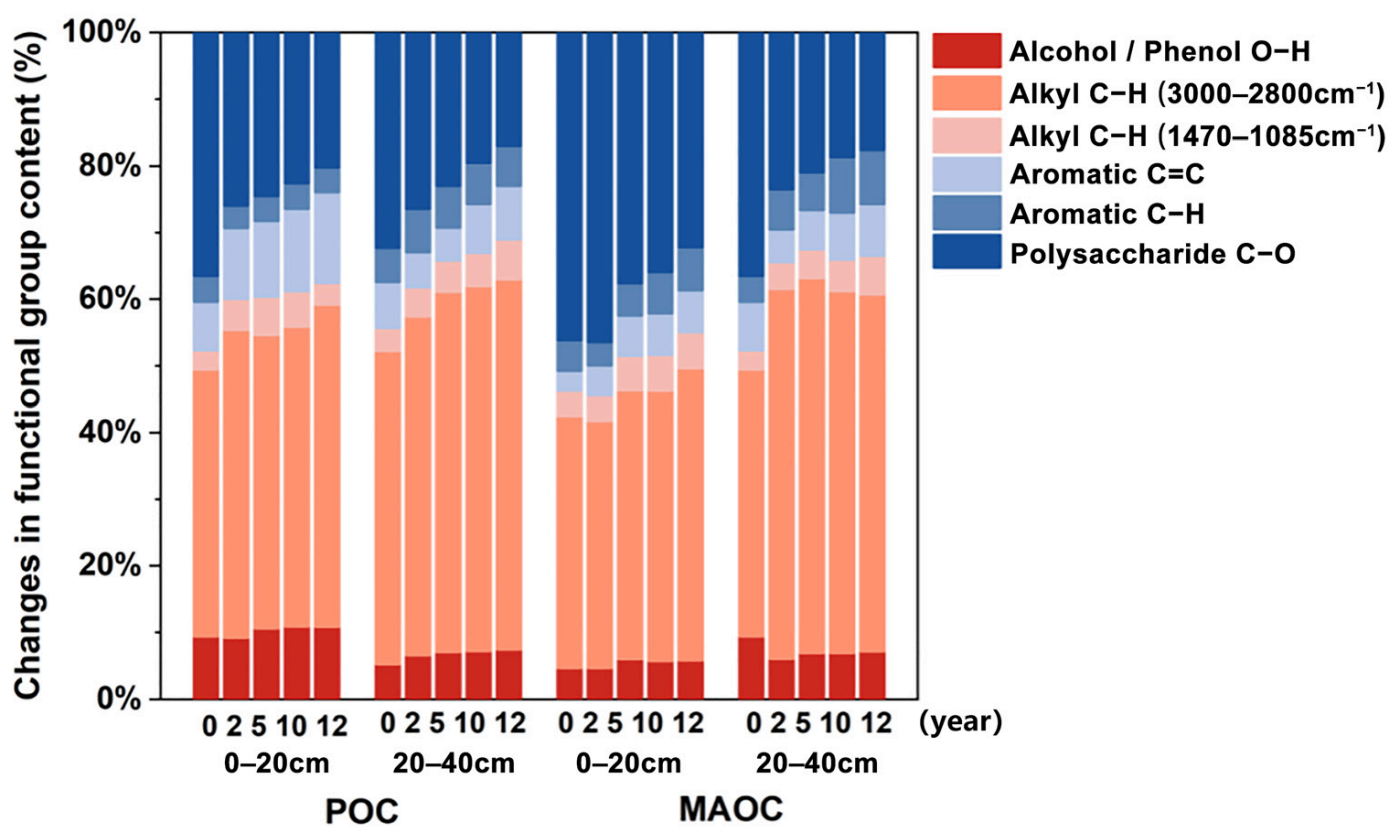
Changes in functional group contents of POC and MAOC in saline–alkaline paddy soils under different rice cultivation durations. Numbers (0, 2, 5, 10, and 12) indicate rice cultivation duration (years). POC (particulate organic carbon); MAOC (mineral-associated organic carbon).

The FTIR spectral absorption peaks trends of POC and MAOC in both soil layers of saline–alkaline paddy soils under different rice cultivation durations were basically consistent. With the extension of cultivation duration, POC and MAOC carbon skeleton structures remained basically unchanged. However, there were certain differences in the absorption intensity of some characteristic peaks (Figure 4).

In terms of functional group composition, the POC in both soil layers was dominated by aliphatic hydrocarbon C-H groups (3000~2800 cm^−1^), accounting for approximately 48.59%, followed by polysaccharide C-O carboxyl groups (about 24.82%), and the proportion of aliphatic hydrocarbon C-H groups (1470~1385 cm^−1^) was the lowest (about only 4.55%). In contrast, for MAOC in the 0–20 cm soil layer, the soils of CK and 2-year cultivation were dominated by polysaccharide C-O carboxyl groups, accounting for approximately 45.95%, while the proportion of aliphatic hydrocarbon C-H groups (1470~1385 cm^−1^) was relatively lower (4.54%), pointing to a higher abundance of carbohydrate-like structures bound to mineral surfaces in early stages.

With the increase in rice cultivation duration, the contents of alcoholic and phenolic O-H hydroxyl groups, aliphatic hydrocarbon C-H groups (3000~2800 cm^−1^), and aromatic alkene C=C groups in POC of each soil layer increased significantly, reflecting progressive accumulation of hydrophobic and aromatic plant residues. Meanwhile, the content of polysaccharide C-O carboxyl groups decreased significantly, demonstrating active microbial decomposition and utilization of labile carbohydrates. For MAOC in each soil layer, the content of aliphatic hydrocarbon C-H groups increased significantly, while the content of polysaccharide C-O carboxyl groups decreased significantly, indicating a compositional shift toward enriched aliphatic structures in mineral-associated pools over time.

#### 3.3.3. Effects of Rice Cultivation Duration on Soil Stability Index of Saline–Alkaline Paddy Soils

**Figure 5:**
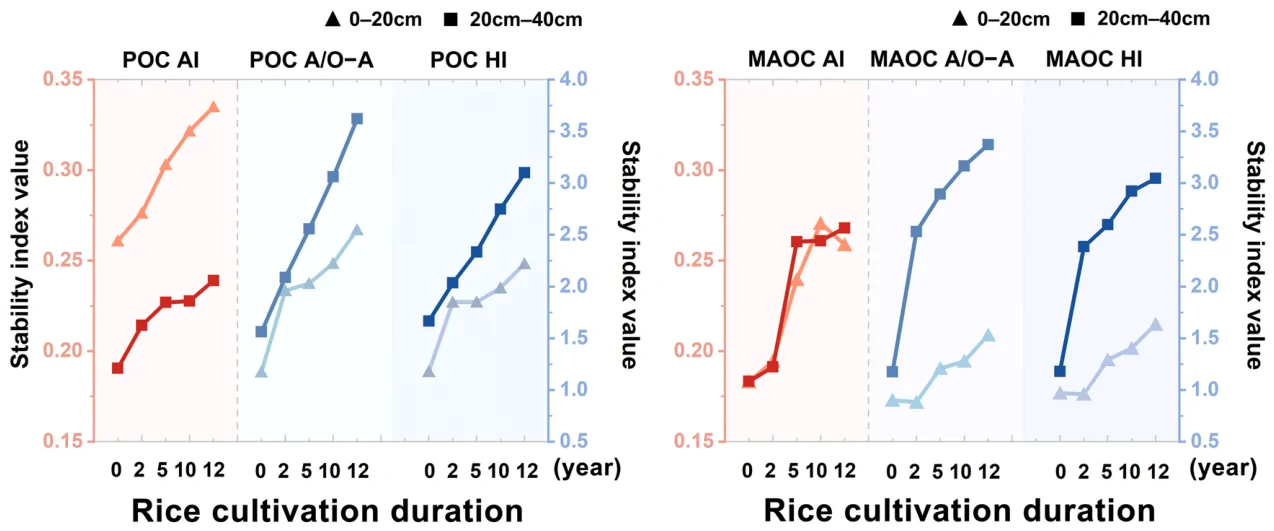
Changes in functional group stability indices of POC and MAOC in saline–alkaline paddy soils under different rice cultivation durations. AI, A/O-A and HI represent the aromaticity index, alkyl carbon/polysaccharide carbon ratio and hydrophobicity index, respectively. Light-colored lines represent the 0–20 cm soil layer, and dark-colored lines represent the 20 cm–40 cm soil layer. Soil layers are primarily distinguished by triangles and squares in the legend.

The stability indices (AI, A/O-A, and HI) of both POC and MAOC showed a significant increasing trend with the extension of rice cultivation duration, reflecting the progressive enhancement of carbon chemical recalcitrance and stabilization over time. The A/O-A and HI indices of POC and MAOC in the 20–40 cm soil layer were significantly higher than those in the 0–20 cm soil layer, and the increase rate of POC was greater than that of MAOC (Figure 5).

The AI of POC in the 0–20 cm soil layer was significantly greater than that in the 20–40 cm soil layer, while the AI of MAOC showed no significant difference between the two soil layers. But, in the 20–40 cm soil layer, the increase rate of MAOC was greater than that of POC, demonstrating accelerated subsurface mineral association processes; specifically, it increased sharply during the 2–5 years of cultivation and tended to stabilize from 5 to 12 years, reflecting a dynamic saturation of mineral surfaces.

### 3.4. Influencing Factors on Soil Organic Carbon Fractions and Their Stability in Paddy Soils with Different Rice Cultivation Durations

**Figure 6:**
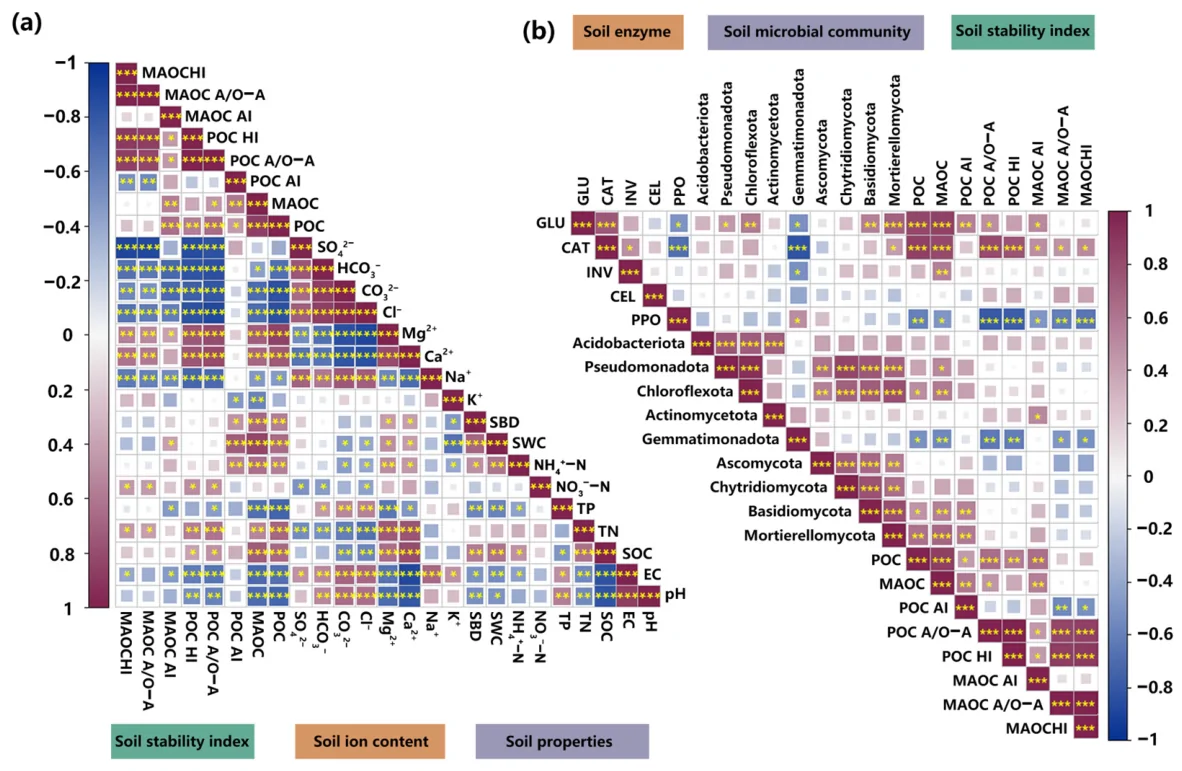
Correlation analysis between soil organic carbon stability indexes and soil factors. (**a**) Correlation between soil properties, soil ions content and soil organic carbon stability indexes. (**b**) Correlation between soil enzymes, soil dominant bacteria and fungi and soil organic carbon stability indexes. In Pearson correlation analysis, *, **, and *** represent significant correlations at the *p* < 0.05, *p* < 0.01, and *p* < 0.001 levels, respectively. AI, A/O-A and HI represent the aromaticity index, alkyl carbon/polysaccharide carbon ratio and hydrophobicity index, respectively. GLU, CAT, INV, CEL and PPO represent *β*-glucosidase, catalase, invertase, cellulase and polyphenol oxidase, respectively.

Correlation analysis indicated that reclamation years, soil organic carbon fractions, soil stability index, soil properties, soil ion content, soil enzyme activities, dominant bacteria, and dominant fungi were significantly correlated (Figure 6). Soil organic carbon fractions and soil stability index were significantly negatively correlated with pH, EC, Na^+^, Cl^−^, CO_3_^2−^, HCO_3_^−^, and SO_4_^2−^ (*p* < 0.001 or *p* < 0.01), and significantly positively correlated with TN, Ca^2+^, and Mg^2+^ (*p* < 0.001 or *p* < 0.01). In contrast, the soil stability index was positively correlated with SOC, SWC, and NH_4_^+^-N (*p* < 0.05). Additionally, soil organic carbon fractions and soil stability index were significantly positively correlated with the soil enzymes *β*-glucosidase (GLU), catalase (CAT), and the dominant soil bacterial phylum Gemmatimonadota (*p* < 0.001 or *p* < 0.05), and significantly negatively correlated with the soil enzyme polyphenol oxidase (PPO) and the dominant soil fungal phyla Basidiomycota and Mortierellomycota (*p* < 0.01 or *p* < 0.05).

### 3.5. PLS-PM Analysis

**Figure 7:**
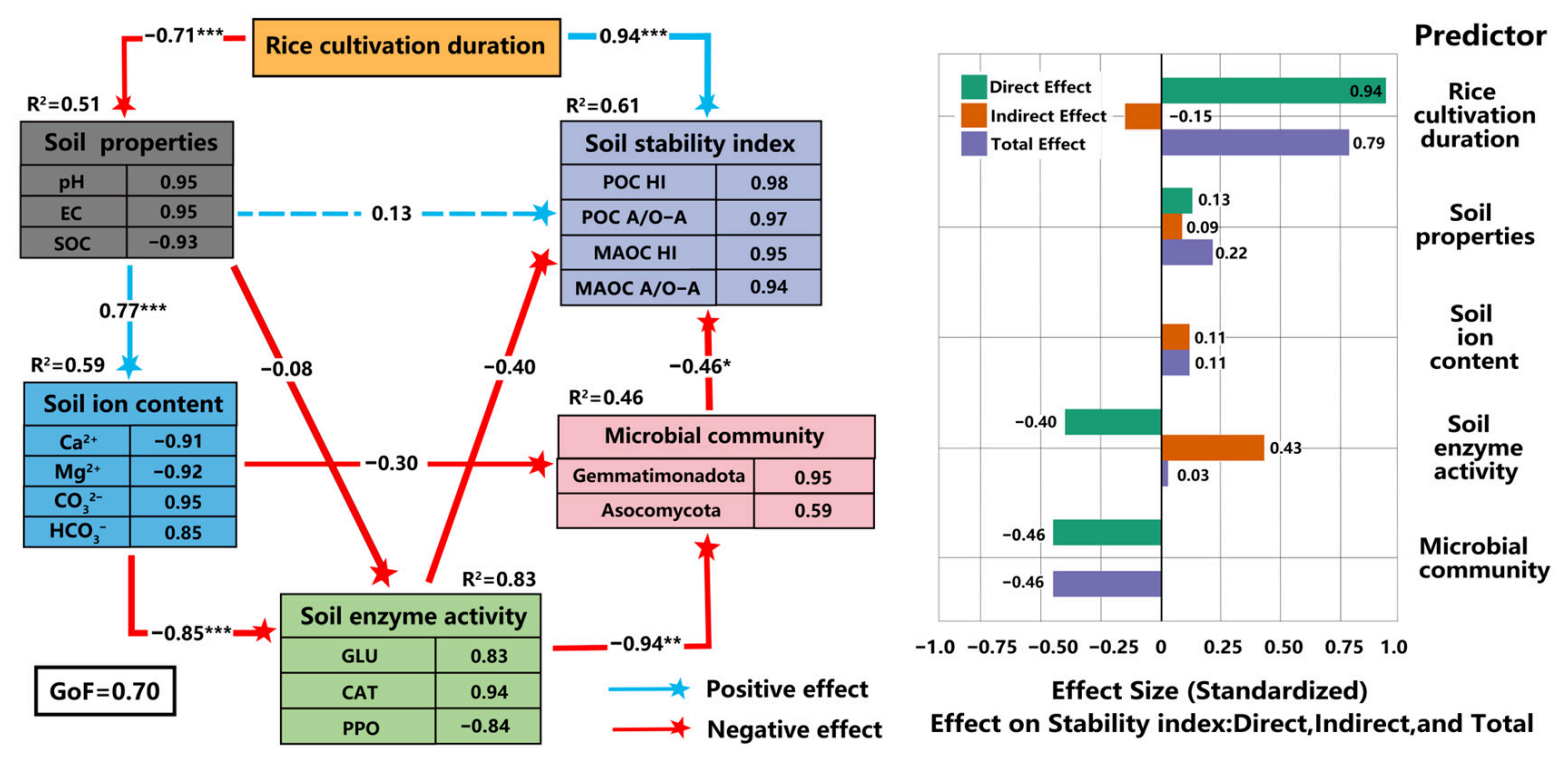
Partial least squares path model (PLS-PM). In the PLS-PM analysis, the width of the arrows is proportional to the strength of the path coefficients, and R^2^ represents the explanatory power of the variables. Red and blue arrows denote positive and negative relationships, respectively, while dashed arrows denote non-significant relationships. *, **, and *** indicate significant effects at the 0.05, 0.01, and 0.001 levels, respectively.

The PLS-PM (Figure 7, GoF = 0.70) was constructed to quantify the interactions among rice cultivation duration, soil physicochemical properties, ion content, microbial community, and enzyme activities, collectively explaining 61% of the variance in soil stability. Rice cultivation duration was the dominant driver, exerting a potent direct positive effect on soil stability (*β* = 0.94, *p* < 0.001), reflecting the cumulative build-up of physical protection and chemical recalcitrance over years of management. However, this was partially offset by a negative indirect effect through a cascading pathway: continuous cultivation significantly altered soil properties (*β* = −0.71, *p* < 0.001), which in turn significantly altered soil ion dynamics and composition (*β* = 0.77, *p* < 0.001). The shift in ion concentrations and proportions substantially inhibited soil enzyme activities (*β* = −0.85, *p* < 0.001) by imposing osmotic and specific ion stress and concurrently shifted the microbial community structure (*β* = −0.30).

Furthermore, soil enzyme activity exerted a direct negative impact (*β* = −0.40) on the stability index—reflecting accelerated organic matter breakdown and turnover—while the microbial community also showed a direct negative effect (*β* = −0.46, *p* < 0.05) linked to community-driven carbon processing dynamics. Conversely, soil properties exhibited a minor direct positive path to stability (*β* = 0.13). As shown in the effect breakdown, the total effect of cultivation duration on soil stability remained strongly positive (*β* = 0.79). These results indicate that rice cultivation duration dictates soil stability through both dominant direct enhancement and complex multi-stage indirect regulation involving soil chemistry and biological activity. In summary, reclamation improves soil physicochemical properties, which alleviates alkalized ion stress and regulates enzyme activities and microbial communities, ultimately enhancing the stability of organic carbon.

## 4. Discussion

### 4.1. Rice Cultivation Progressively Improves the Physicochemical Environment of Saline–Alkaline Paddy Soils

Continuous rice cultivation markedly improved the physicochemical environment of saline–alkaline paddy soils, as evidenced by coordinated decreases in soil pH, EC, and SBD, together with the continuous increases in SOC and nutrient contents. Soil pH, EC, and SBD are widely recognized as key indicators for evaluating the degree of soil salinization and the effectiveness of reclamation practices [40]. These coordinated changes indicate that continuous rice cultivation progressively alleviated saline–alkaline stress and established a favorable soil environment for nutrient cycling and SOC stabilization.

The rapid decreases in soil pH and EC during the first 2–5 years of cultivation indicate that flooding irrigation effectively accelerated the leaching of soluble salts from the root zone, representing the initial stage of saline–alkaline soil reclamation. Similar trends were reported by Shi et al. [41] and Yang et al. [42]. The downward migration of Na^+^ and Cl^−^ from the 0–20 cm layer to the 20–40 cm layer further confirms that water-driven leaching dominated the redistribution of soluble salts, which is consistent with the observations of Yang et al., Zhou et al. [43,44] and Roth et al. [45].Although prolonged rice cultivation significantly moderates sodicity, a drastic pH decline within a short timeframe cannot be achieved by plant uptake alone, given the negligible initial exchangeable calcium pools under high-pH regimes. Instead, this rapid alkalinization mitigation relies heavily on the synergistic input of agricultural amendments and mineral fertilizers, which supply exogenous protons and divalent cations to accelerate carbonate dissolution and facilitate the displacement of exchangeable sodium. Meanwhile, the continuous enrichment of Ca^2+^ and Mg^2+^, particularly in the plow layer, suggests that reclamation involved not only physical salt removal but also gradual improvement in soil ionic balance through cation exchange. Similar mechanisms have been proposed by Lv et al. [46] and Zhou et al. [47], who demonstrated that Ca^2+^ and Mg^2+^ play essential roles in alleviating sodicity and restoring soil structure. Although EC continued to decrease with cultivation duration, soil pH stabilized after approximately 10 years [48], suggesting that reclamation gradually reached a relatively stable physicochemical equilibrium maintained by the buffering capacity of carbonate and bicarbonate systems.

The improvement in ionic balance was accompanied by progressive optimization of soil physical structure, as reflected by the continuous decrease in SBD and increase in SWC. Zhu et al. [49] and Zhang et al. [50] proposed that approximately five years of cultivation represents a critical transition stage, during which SOC accumulation of becomes the dominant driver of soil physical remediation. Similar to the beneficial effects of straw incorporation [51], continuous inputs of rice residues promoted aggregate formation, improved pore connectivity, and enhanced soil water retention, thereby alleviating soil compaction. These structural improvements provide favorable conditions for root development, microbial colonization, and microbial carbon transformation, ultimately facilitating SOC stabilization. Specifically, prolonged cultivation stimulates root proliferation and active exudation within the ameliorated matrix, supplying essential organic substrates that selectively enrich rhizosphere microbes and drive nutrient cycling (such as nitrogen transformations) [52]. These root–microbial interactions accelerate the breakdown and stabilization of organic residues, forming a synergistic feedback loop between plant roots, microbial communities, and nutrient availability that underpins long-term carbon sequestration in reclaimed paddies [53].

In contrast to upland black soil reclamations in Northeast China, which often experience an initial sharp drop in SOC during the first 3 years of cultivation driven by intensive tillage-induced soil disturbance and aggregate disruption [16,17], our saline–alkaline paddy chronosequence showed an immediate, steady upward trajectory in carbon stocks without a preliminary depletion phase. This discrepancy highlights that the unique hydrodynamic and anaerobic conditions of paddy fields buffer against the severe carbon losses typical of dryland tillage conversions, allowing for root-derived carbon inputs to immediately outpace decomposition losses even in early reclamation stages.

The synchronous increases in SOC, TN, TP, and NH_4_^+^-N indicate that rice cultivation gradually transformed saline–alkaline soil from a nutrient-deficient system into a relatively balanced ecosystem with enhanced nutrient cycling and carbon storage capacity. The progressive amelioration of soil fertility and nutrient stoichiometry is fundamentally maintained by anthropogenic inputs versus crop export dynamics, wherein regular basal and topdressing fertilization, coupled with enhanced biological nitrogen fixation under submerged paddy conditions, effectively compensates for nutrient removal through crop harvest. Compared with the nutrient stratification observed in uncultivated soil [54], long-term rice cultivation promoted a more homogeneous nutrient distribution through tillage and continuous flooding [55]. Similar responses have also been reported by Iqbal et al. [56], who demonstrated that long-term nutrient inputs regulate microbial activity and soil biogeochemical processes. Our results further support the positive feedback mechanism proposed by Brahim et al. and Qu et al. [13,57], whereby SOC accumulation enhances nutrient retention, while improved nitrogen and phosphorus availability promotes plant biomass production and continuous carbon inputs. Collectively, the progressive improvement in soil physicochemical properties established the environmental basis for subsequent changes in SOC fractions and molecular structure, which ultimately regulate SOC stability.

### 4.2. Rice Cultivation Promotes SOC Stabilization Through Changes in Carbon Fractions and Molecular Structure

Following the progressive improvement in soil physicochemical properties, long-term rice cultivation further enhanced SOC stability by regulating carbon fraction distribution and molecular structure. The continuous increase in the proportion of MAOC, particularly in the 0–20 cm soil layer, indicates that improved soil conditions favored the stabilization of plant-derived carbon through mineral association, supporting the mineral protection mechanism of SOC stabilization [58,59]. In contrast, the relatively limited accumulation of MAOC in the 20–40 cm layer was likely constrained by lower oxygen availability and reduced substrate inputs. Meanwhile, the increasing MAOC/POC ratio reflects a gradual shift from particulate organic carbon toward mineral-associated carbon dominance [60], a process widely recognized as an indicator of enhanced SOC persistence and long-term carbon sequestration [61]. Furthermore, the biogeochemical dynamics governing carbon fractions across surface and deeper soil layers are profoundly influenced by microbial diversity and functional potential, which are closely related to the vertical stratification and abundance of genes involved in nutrient cycling [62].

Rapid MAOC accumulation observed in this study differs from findings in coastal saline–alkaline and highly sodic alluvial soils, where high exchangeable Na^+^ and dispersed clay were assumed to hinder organo-mineral interactions [18]. While high sodium is conventionally believed to disperse colloids and suppress microaggregate and MAOC formation according to DLVO theory, the progressive substitution of Na^+^ by Ca^2+^/Mg^2+^ and intensive rice root exudation surmounted this chemical barrier. This result demonstrates that rice-mediated biological amelioration can effectively overcome physicochemical constraints on mineral saturation.

The redistribution of SOC fractions was accompanied by pronounced molecular transformation. The enrichment of alcoholic and phenolic O–H groups together with aromatic C=C structures suggests the continuous incorporation of plant-derived polyphenols and aromatic compounds into both POC and MAOC [63]. These compounds facilitate humification and strengthen organo-mineral associations, thereby enhancing carbon stabilization [64]. Conversely, the decline in aliphatic C–H and polysaccharide C–O groups indicates the preferential decomposition of labile carbon fractions and the relative enrichment of chemically resistant components [65]. The increased aromaticity is primarily driven by the accumulation of recalcitrant plant-derived residues (such as lignin and polyphenols) and selective microbial resynthesis, whereas the decrease in polysaccharides stems from their rapid microbial utilization as a primary energy source during active soil amelioration and organic matter turnover [53]. This interpretation is further supported by changes in soil biological processes. Enhanced *β*-glucosidase (GLU) and catalase (CAT) activities [66], together with reduced polyphenol oxidase (PPO) activity [67], favored the conversion of labile carbon into more stable aromatic compounds while suppressing the degradation of lignin-derived carbon [68]. Concurrent shifts in the microbial community, particularly the reduced abundance of Gemmatimonadota and Ascomycota, further suggest that the alleviation of saline–alkaline stress reshaped microbial carbon utilization and promoted the incorporation of microbial residues into MAOC [69]. Collectively, these biological processes provide a mechanistic explanation for the increasing MAOC/POC ratio and the enrichment of aromatic carbon components, which is consistent with the molecular preservation framework proposed by Keiluweit et al. [70].

Overall, long-term rice cultivation promoted SOC stabilization through the coordinated regulation of carbon fractions, molecular structure, and biological processes [71]. The stronger responses observed in the 0–20 cm layer reflect greater root-derived carbon inputs, whereas similar but less pronounced trends in the 20–40 cm layer indicate that the mechanisms of SOC stabilization operated consistently throughout the soil profile [72,73]. These findings emphasize that sustained organic matter inputs and favorable mineral protection conditions are essential for promoting the transformation of labile carbon into stable MAOC during saline–alkaline paddy soil reclamation [74]. Together, these results provide the mechanistic basis for the integrated pathways of SOC stabilization discussed in the following section.

### 4.3. Integrated Mechanisms Underlying SOC Stabilization During Long-Term Rice Cultivation

The PLS-PM integrated the physicochemical and biological processes identified throughout this study into a unified mechanistic framework, demonstrating that SOC stabilization during saline–alkaline paddy reclamation was regulated by coordinated interactions rather than by individual environmental factors. Rice cultivation duration acted as the primary driving force, exerting both direct and indirect effects on SOC stability through the sequential regulation of soil physicochemical properties, biological processes, and carbon transformation. This integrated pathway agrees well with previous conceptual frameworks by Nie et al. [75], describing SOC stabilization as the outcome of coupled environmental and biological controls rather than isolated mechanisms.

The results suggest that SOC stabilization followed a clear stage-dependent trajectory during long-term rice cultivation. In the early reclamation stage, flooding irrigation and salt leaching rapidly alleviated saline–alkaline stress, resulting in improved soil structure, reduced ionic constraints, and enhanced nutrient availability. As soil conditions became progressively more favorable, biological regulation became increasingly important. Changes in enzyme activities and microbial community composition promoted the transformation of plant-derived residues into mineral-associated organic carbon, accompanied by the enrichment of chemically stable aromatic structures. The coordinated evolution of soil environment, microbial processes, and carbon chemistry is consistent with the molecular preservation framework proposed in previous studies, emphasizing that long-term SOC persistence depends not only on carbon input, but also on the efficiency with which microbial products are stabilized through mineral association [16].

Overall, our findings indicate that the stabilization of SOC in saline–alkaline paddy soils is a progressive process driven by the interaction of physicochemical improvement, biological regulation, and mineral protection rather than by any single factor. This mechanistic framework not only explains how long-term rice cultivation enhances SOC stability but also provides a theoretical basis for optimizing saline–alkaline land reclamation. Maintaining continuous organic matter inputs while improving soil physicochemical conditions is essential for promoting the formation and preservation of MAOC, thereby maximizing long-term carbon sequestration potential in saline–alkaline paddy ecosystems [76].

While this study provides valuable insights into carbon stabilization in reclaimed sodic soils, the generalizability of these findings is subject to specific environmental boundary conditions. The observed cascade mechanisms and MAOC accumulation rates closely reflect the unique temperate continental monsoon climate and specific parent materials of Jilin Province. Consequently, these specific temporal thresholds and quantitative trends may differ in other saline–alkaline environments—such as coastal saline soils or arid inland regions—where distinct hydro-thermal regimes, initial sodicity levels, and soil mineralogies could alter microbial decomposition rates and organo-mineral interactions.

## 5. Conclusions

This 12-year chronosequence study demonstrates that long-term paddy management progressively enhances soil fertility and drives organic carbon sequestration through a cascade-driven mechanism of physicochemical amelioration, biological regulation, and carbon molecular transformation. Specifically, after 12 years of cultivation, EC in the 0–20 cm layer decreased significantly by 70.11%, while SOC and TN increased by 63.09% and 26.02%, respectively. Furthermore, MAOC accumulated rapidly, increasing by 147.12% in the topsoil and shifting the carbon pool toward structural persistence. Partial least squares path modeling further revealed that cultivation duration acted as the dominant direct driver of soil stability (*β* = 0.94, *p* < 0.001), operating through a cascading pathway where management practices significantly modulated soil enzyme activities and microbial communities, which in turn accelerated the conversion of labile carbon into mineral-associated and aromatic-rich stable pools. While these findings provide robust evidence for the trajectory of carbon stabilization in reclaimed sodic soils, several limitations should be noted: the study relies on a space-for-time chronosequence rather than long-term continuous tracking of identical plots, and it does not explicitly quantify the specific contribution of exogenous organic amendments versus root-derived inputs. Future research should prioritize long-term field monitoring combined with isotopic tracing techniques to better differentiate carbon sources and evaluate the sustainability of carbon sequestration under changing climatic and management scenarios.

## Figures and Tables

**Table 1:** Effects of different sources of variation on soil organic carbon fractions.

Index	Source of Variation	df	Mean Square	F-Value	*p*-Value	Effect Size
POC	Year	4	17.489	43.002	<0.001 ***	0.896
	Layer	1	0.137	0.337	0.568	0.017
	Year × Layer	4	5.259	12.929	<0.001 ***	0.721
MAOC	Year	4	35.233	67.661	<0.001 ***	0.931
	Layer	1	18.593	35.706	<0.001 ***	0.641
	Year × Layer	4	6.153	11.816	<0.001 ***	0.703

Notes: *** represent significant correlations at the *p* < 0.001 levels.

## Data Availability

The research data supporting the conclusions of this study are not publicly available at present. This is because the data set will be utilized for subsequent extended research work, so as to ensure the continuity of the relevant research and the consistency of in-depth data analysis. For legitimate academic inquiries that comply with ethical and academic norms, interested parties may contact the corresponding author (DanZhang, zhangdan@ccu.edu.cn) after the completion of the follow-up research to request access to the data upon appropriate review and approval.

## Abbreviations

The following abbreviations are used in this manuscript:

SOC	Soil Organic Carbon
POC	Particulate Organic Carbon
MAOC	Mineral-Associated Organic Carbon
AI	Aromaticity Index
A/O-A	Alkyl carbon/polysaccharide carbon ratio
HI	Hydrophobicity Index
PLS-PM	Partial Least Squares Path Model
FTIR	Fourier Transform Infrared Spectroscopy
EC	Electrical Conductivity
SBD	Soil Bulk Density
SWC	Soil Water Content
TN	Total Nitrogen
TP	Total Phosphorus
NO_3_^−^-N	Nitrate Nitrogen
NH_4_^+^-N	Ammonium Nitrogen
GLU	*β*-Glucosidase
CAT	Catalase
INV	Invertase
CEL	Cellulase
PPO	Polyphenol Oxidase
